# Advancing regionalism and a social policy agenda for positive change: From rhetoric to action

**DOI:** 10.1177/1468018115600123

**Published:** 2015-12

**Authors:** Viviene Taylor

**Affiliations:** University of Cape Town, South Africa

## Introduction

In the context of deepening poverty, growing inequalities within and between countries and the uneven impacts of economic and financial globalisation, we need to consider how social development, underwritten by a people-centred approach, can influence existing and emerging regional processes and change the regional social policy agenda. Moreover, there is a new urgency given the scale of poverty and social exclusion experienced in countries in Africa and in the global south, to move away from the belief that regionalism both as a policy agenda and as a development project should be limited only to issues of economic integration, trade negotiations and the pursuit of national and regional security.

## What kind of regionalism for a social policy agenda?

The concept of regionalism and associated processes is part of on-going debate and contestation especially when it comes to who benefits from these processes and under what conditions. Some years ago, Nye defined a region ‘as a limited number of states linked by a geographical relationship and by a degree of mutual interdependence’, and he refers to (international) regionalism as ‘the formation of interstate associations or groupings on the basis of regions’ (Nye, 1968: vii). This way of looking at regionalism assumes that states linked by common geographical boundaries would come together to address issues of mutual interest. It ignores the historical, political, social and economic conditions that influence processes of change within and external to states. These conditions, especially in Africa, are outcomes of colonial and post-colonial struggles which influenced liberation and state formations in many different ways. The ‘balkanisation’ of Africa as an outcome of colonial tradeoffs and the re-inscribing of new forms of economic colonialism makes it particularly difficult for countries to adopt regional systems of governance, regional cooperation, regional trade integration and regional social policies. Despite this reality, the issues and conditions that demand social policy action in Africa require us to think differently about regionalism and its significance for the development of the poorest and most disaffected people.

Regionalism in the sense that I use it in this article is more than a geographical location of states with common boundaries. It is also more than a constellation of states seeking to develop economic and trade benefits, while such states try to influence the balance of power between those considered super powers and emerging powers. Regionalism for positive social change is a process that is socio political in forging a new social policy agenda that focuses on addressing some of the most critical social conditions affecting the majority of poor and disenfranchised people in the continent and in the global south. Such a social policy agenda has to emerge through mobilisation of people’s movements from below and connect with regional institutions and networks of formal policy processes. The critical challenge therefore confronting many of our countries is how to develop a type of regionalism that will strengthen and at the same time diversify cooperation in ways that will ensure benefits for people within states as well as for regions experiencing common and unique social challenges.

## Learning from history

The genesis of regionalism in the south goes back to after the Second World War and was shaped by the liberation and anti-colonial movements’ idea of collective self-reliance. The Afro-Asian Conference at Bandung in 1955, the founding of the Non Aligned Movement (NAM) in 1961 and the Group of 77 in 1964 were some of the early initiatives to develop a process that would ensure collective action for common regional challenges and concerns. The emergence of South–South links in the 1960s in Latin America and the Caribbean, through the Latin American Free Trade Association and the Caribbean Community, were also attempts to expand development or economic opportunities, in the face of on-going economic marginalisation and a hegemonic agenda of western industrial countries.

The Southern African Development Community (SADC) is a variant of the European Union (EU) model of regionalism. Unlike the historic NAM launched in Bandung in Indonesia by 115 representatives from Africa and Asia whose thinking at the time was to counter the hegemony of western countries, SADC’s primary focus is the promotion of regional economic integration. NAM’s (n.d.) concern at that time was to promote a development agenda by maintaining political independence and by opposing forms of colonialism, neo- colonialism and western dominance. While some of this history may inform emerging regional groupings, the changed geopolitical landscape and the social impacts of globalisation also significantly influence regional alignments.

## The changed geo-political environment

New alignments among regional and global institutions of, largely, economic governance creates complex challenges for a social policy agenda. Globalisation creates new opportunities and generates greater risks as national borders become permeable and the traditional role of the nation state is challenged. At the same time, processes of social development and poverty eradication depend on the extent to which states are able to manage the process of global integration in the interests of the majorities of poor people. Furthermore, it depends on how social policy issues are put onto the regional agenda.

There are challenges in navigating and establishing a regional social policy agenda. Countries in Africa and indeed in the south are trying to proactively position themselves in a ‘globalised world with uni-polar characteristics’. Evident in this positioning is the recognition that the level of complexity in the type of relationships required at a bilateral and multilateral level demands a better understanding of the strategic social policy or social development challenges facing countries and regions (Republic of South Africa: Department of Foreign Affairs [RSA], 1999: 3).

Central among these challenges is how to locate regional initiatives in a world that has undergone a shift from a *bipolar* East–West Cold War dichotomy (or *tripolar* configuration, if the third world is included), to one in which there seems to be only one global hegemonic power (Huntington, 1999: 35). In this context, the challenge is to find critical spaces to engage in a regional and global arena in ways that would promote both national interests that focus on people’s development as well as consolidate a relationship within the global economic south to ensure inclusive, pro-poor development.

## Regionalism within Africa

Overlaying Africa’s status as a regional power is the reality of globalisation. The worldwide spread of industrial production and new information technologies accompanied and promoted by the rapid and unimpeded mobility of capital, unfettered free trade, the global reach and authority of transnational corporations and the digitalisation of money has serious implications for social development. This process – concentrated in an essentially Euro-American (except for Japan) condominium of post-industrial economies – forms the core of the global system (Marais, 1998). The relative or strategic importance of states is also a factor in the realignments that shape emerging power blocs (Taylor, 2000). As one of Africa’s pre-eminent economic powers, South Africa, by some definitions, is also considered a ‘*pivotal* state’ (Economist Review, 1999: 9). For example, the SADC beginnings in 1980 were in part a political response to counter the dominance of former apartheid South Africa. Post-1994 with the emergence of democratic rule in South Africa, this original focus was changed to reflect a focus on economic integration processes which are supported by regional integration agreements (RIAs). Proponents of integration generally assume that the benefits of regional economic integration will result in new markets and improve economic growth prospects (South African Institute of International Affairs, 2005). Yet economic growth in countries in Africa has not translated into better human development outcomes for the poorest peoples. The uneven impacts of economic globalisation spurred by a neoliberal agenda have not resulted in reduction in inequalities and social development.

Reconceptualising poverty eradication strategies within an African regional agenda that is part of the African Renaissance project could provide political and moral legitimacy for such social policy interventions. The African renaissance strategy includes the development of a national and sub-regional programme. As an initiative, it provides scope for networking and exchanges with African researchers and professionals throughout the Continent. It is a programme that also embraces an outreach to the African diaspora, a dimension that could coordinate an African renaissance agenda with South–South co-operation strategies. Can a regional initiative such as the ‘African Renaissance’ chart the way for pan-Africanism revisited with a social policy agenda as part of its goals? Key to these issues is whether countries proceed along a path of *competitive regionalism* or *cooperative regionalism with a progressive social policy agenda*, especially within the geopolitical ‘Cape to Cairo’ axis of eastern and southern Africa. This will depend on the extent to which sub-regions within the continent consciously link social and economic goals to address structural conditions that deepen poverty and increase inequalities.

Has regional economic integration and the establishment of regional institutional arrangements such as the African Union made a difference in how states pursue a social policy agenda on the continent? Since the establishment of a Social Policy Framework for Africa ratified by Ministers of Social Development within the African Union (2008), more emphasis is being given to developing coherent social policies. Social programmes within countries are beginning to align with such social policies. The evidence in the region does not however reflect the policy rhetoric of African states when it comes to promoting the AU’s social policy framework. A review of African states’ performance in achieving the Millennium Development Goals is disappointing and confirms the gap between the policy rhetoric and programme implementation. Disappointingly 25 out of 50 countries still have very high poverty levels with more than 25% of the population in these countries living under US$1.25 per day. The proportion of the population experiencing high hunger is between 25% and 35% in 14 countries. Alarming indicators of child mortality exist in the region with very high mortality rates (more than 150 deaths per 1000 children under the age of five) in 8 countries and high mortality (between 80 and 150 deaths per 1000 children under the age of five) in 20 countries. Maternal mortality indicators are also very high in 25 countries with 200–500 maternal deaths to 100,000 live births. The HIV incidence rate of new HIV infections per year per 100 people aged 15–49 is more than 25% in 20 countries (United Nations, n.d.).

Given these trends we need to ask whether a regional social policy agenda facilitate the development of state capacity to design and manage policies and programmes to promote human development through sub-regional institutions and national structures. Importantly, can a regional agenda for social development evolve from concerted dialogue on critical issues identified by AU member countries within the context of regional and country specificity? The emergence of a Social Policy Framework in the African Region is an advance but this framework needs to inform the agenda for economic integration by focusing on the social impacts of economic and political crises.

## Some principles to guide a regional agenda for social development

Social development within the regional context is defined as an integrated holistic process of social and economic change that results in improvements in the lives of all while prioritising the poorest. Captured in the definition is the aim of improving the quality of life of all, particularly those who are excluded from mainstream society as a result of poverty, gender discrimination, unemployment, racism and civil conflicts among others.

A regional agenda for social development requires a focus on the well-being of all members of society with particular emphasis accorded to those who are disadvantaged and vulnerable. The promotion of human rights, democracy, transparent and accountable governance alongside the active participation of citizens and civil society organisations in the design and implementation of a regional social development agenda should be made explicit. If Africa is to translate the social policy framework into reality and advance the post-2015 sustainable development goals (SDGs), then some principles that emerge from lessons of experience are worth noting.

First, regional cooperation should be based on more than the current trend of securing markets for economic growth and security. There should be a concerted, systematic attempt to develop national and regional consciousness on the links between macro-economic concerns and social policy imperatives. Second, the promotion of democracy, peace and human security alongside an active regional agenda to address poverty and HIV/AIDS must be central to a regional strategy for social development. Third, regional social development initiatives require effective coordination, technical and financial resources as well as institutional space and capacity. Fourth, urgent steps need to be taken to ensure capacity building and effective use of development aid on common problems in the region. Capacity building is not only required for officials but also at the level of political and community leadership.

Fifth, a regionally integrated human development strategy calls for shared responsibility with the broad range of civil society organisations and the private sector. Such a strategy has to be based on country and regional needs to address short- and long-term development goals. The participation of community representatives and organisations in new partnerships with governments and business could be enhanced through the provision of financial assistance and by identifying connecting points for such engagement. Furthermore, the tendency to substitute issues of distribution (power and resources) with the need for efficient management within the public and non-governmental sectors is cause for concern. In developing shared responsibility for transformation, it is important for all partners to understand that different sectors bring different but critical qualities to the process. The role of governments cannot be substituted by civil society organisations or by market forces on the basis of efficiency arguments.

Sixth, attention has to be given to the need to improve information flows and communication processes in the region. This is not only in relation to official processes but also with the full range of civil society organisations. Ensuring that citizens are empowered with appropriate information and knowledge to access their rights and entitlements is an imperative in building a people-driven social development process. Consolidating regional relations within a human rights framework to address social development is also vital. A particular challenge for the region is ensuring that a progressive agenda of internal and regional change will be located within a human rights framework.

In recasting a regional role for social development in the global system, countries have the complex task of negotiating both spaces and agendas that will ensure political credibility, moral legitimacy and a people-centred development path that is sustainable and socially just. The challenges therefore are many. They include the urgent need to satisfy the popular expectations raised through international commitments. Effective ways must be found to mediate the wide range of competing political, social and economic pressures that continue to be advanced by different social forces in our countries. There is a need to work together to reconcile the almost universal tension between the internal needs of bureaucracy and the needs of citizens, in order to accelerate service provision within a caring and enabling framework. There is also a need to negotiate the difficult path between political democratisation and the dominance given to economic liberalisation.

Moreover, governments need to resolve the discontinuity between policy objectives and outcomes. In doing this, they need to distinguish more clearly between ineffective implementation (resulting from a lack of capacity, particularly funds, rather than a commitment to the implementation of government policy) and non-implementation (resulting from a lack of compliance with such policy). As efforts are made to implement regional agendas for social development, it will also be important to devise appropriate and differentiated strategies to address these two issues.

The need for more effective forms of partnership between states and civil society has been bolstered by informed research (Evans, 1996; Nye, 1968; Rodrik, 1996; The World Bank, 1997) which has demonstrated that the increased capacity of states is positively, rather than inversely, correlated with the increased vitality of civil society. As civil society grows more robust, the capacity of states to govern is increased. This is vital for the building of forms of regionalism from below and that has its basis in social solidarity and subsidiarity.
